# Anal canal cancer treatment: practical limitations of routine prescription of concurrent chemotherapy and radiotherapy

**DOI:** 10.1038/sj.bjc.6601378

**Published:** 2003-11-25

**Authors:** L Chauveinc, X Buthaud, M C Falcou, V Mosseri, A De la Rochefordière, J Y Pierga, J Girodet, R J Salmon

**Affiliations:** 1 Radiotherapy Department, Institut Curie, 26 rue d'Ulm, 75248 Paris, Cedex 05, France; 2 Statistics Department, Institut Curie, 26 rue d'Ulm, 75248 Paris, Cedex 05, France; 3 Chemotherapy Department, Institut Curie, 26 rue d'Ulm, 75248 Paris, Cedex 05, France; 4 Surgery Department, Institut Curie, 26 rue d'Ulm, 75248 Paris, Cedex 05, France

**Keywords:** anal carcinoma, radiotherapy, chemotherapy, toxicity

## Abstract

This study is an analysis of the criteria considered when prescribing concomitant chemotherapy and radiotherapy, as a routine treatment for patients with anal canal cancer, and related complications. Between 1990 and 1996, 67 patients were treated at Institut Curie for invasive, nonmetastatic cancer of the anal canal. Median age was 65 years (range, 35–90 years). TNM stage distribution was as follows: seven T1, 17 T2, 27 T3, 16 T4, and 22 N+ patients. A total of 29 patients (i.e., five T1/T2, and 24 T3/T4) received concurrent chemotherapy and radiotherapy. Radiotherapy volumes and dose and prescribed dose for chemotherapy were not statistically different from one group of patients to another. Only 55% of T3/T4 patients underwent standard chemoradiation treatment for anal canal cancer. Age was the one of main factor in determining if the patient would undergo concomitant chemotherapy or not. For the T3/T4 patients, concomitant chemotherapy was prescribed to 69% of patients <55 years, 90% of patients between 56 and 64 years, 45% of patients between 65 and 75 years, and 20% of patients over 75 years (*P*<0.02).Overall survival at 4 years was 66%. The 4 years overall survival rate of T3/T4 patients, who underwent concomitant chemotherapy, was 72%, and that of T3/T4 patient who did not, was 34% (*P*<0.04). The patients who did not undergo chemotherapy were significantly older. The difference in cause-specific survival rates (72 *vs* 48%) was not significant. Relapse-free interval without local recurrence at 4 years was 70%. Relapse-free interval of T3/T4 patients was 78% with chemotherapy and 60% without chemotherapy (*p*=NS). Rates of treatment discontinuation and early toxicity were not statistically different. Late complications occurred in 33 patients, eight of whom had grade 2/3 tumours. At 2 years, complications occurred in 39% of patients who had undergone concomitant chemotherapy, and in 20% of patients who had not (*p*<0.02). Differences in grade 2/3 complications were not significant. In conclusion, although radiotherapy with concomitant chemotherapy is considered the current ‘gold-standard’ treatment for anal canal cancer, in our daily experience, only 55% of our T3/T4 patients have undergone this treatment. The remainder did not undergo chemotherapy mainly because they were deemed too old. In this series, no increase in local control and cause-specific survival was observed in patients who received concomitant chemotherapy; this may be due to the small number of patients included in the series. The increased rate of late complications observed in patients who received the combined treatment, however, provides evidence that this treatment should be restricted to younger patients without comorbidity and therefore justifies our position. Perhaps reduction of doses of chemotherapy must be discussed for older patients.

Carcinoma of the anal canal is a relatively uncommon tumour (1–3% of all cancers of the lower digestive tract) (Beahrs, 1985). The standard treatment for anal cancer is concurrent chemotherapy and radiotherapy. Two publications, reporting on randomised trials of concurrent chemotherapy and radiotherapy, showed the advantages of this association over radiotherapy alone, in terms of local control and colostomy-free (UKCCCR, 1996; Bartelink *et al*, 1997). In these papers, however, the combined treatment was mainly reserved for T3/T4 or N+ patients.

In our experience, patients with T3/T4 and N+ disease are not so frequent. For instance, in a series of 346 patients treated for anal canal cancer at our institution between 1967 and 1996, 33% had a T3/T4 tumour and 23% had an N+ tumour (Chauveinc *et al*, 2003). If concurrent chemotherapy and radiotherapy is the ‘gold standard’ of treatment for large tumours, this treatment was no validated for the smaller-sized tumours.

In this study, we analysed the criteria considered when determining which treatment should be prescribed (concurrent chemotherapy and radiotherapy or radiotherapy alone), the associated results, and complications. This study conducted at Institut Curie includes 67 patients treated for anal canal cancer with 5FU/cisplatin concurrent chemotherapy and radiotherapy, between 1990 and 1996.

## PATIENTS AND METHODS

The sample consists of 67 patients with locally advanced nonmetastatic anal canal cancer who were treated at our institution between 1990 and 1996. The sample excluded patients with tumours of the anal margin, adenocarcinomas, and melanomas. Pretreatment evaluation included history, physical examination, chest radiography, tumour biopsies, standard laboratory tests, and ultrasound (US) or CT-based evaluation of the liver and lymph nodes. Endorectal ultrasound was performed on a small number of patients (the most recent cases only), therefore the results of this test will not be considered in this study. The median duration of follow-up was 48 (10–101) months.

Tumour characteristics and treatments are summarised in Table 1

**Table 1:** Patient characteristics

	**Total**	**Chemotherapy**	**Without chemotherapy**	***P***
Number of patients	67	29	38	
Mean age (years)	65	60.7	67.9	<0.01
Age (years)				
⩽65	35	19	16	
66–75	23	8	15	<0.03
>75	12	2	10	
Pelvic irradiation	1	0	1	NS
Pelvic surgery				
11	2	9	NS	NS
Stage T1/T2	24	5	19	<0.006
Stage T3/T4	43	24	19	
N+ (total)	22	13	9	
Inguinal	9	7	2	<0.04
Pelvic	16	9	7	NS
Squamous differentiated	49	17	32	=0.064
Squamous undifferentiated	15	10	5	NS
Transitional	3	2	1	
Circumference				NS
⩽1/2	49/66	19	30	
>1/2	17	9	8	
Mean high *T* (cm)	4.4/65	5.34	3.63	<0.001
Tumour size	/65			<0.05
⩽4	34	8	26	
>4	31	21	10	
Dose, anal canal (Gy)		62.6	62.2	NS
Brachytherapy	13	4	9	NS

NS=nonsignificant. *P*=*P*-value.

. The Rousseau staging system was used (Table 2

**Table 2:** TNM classification system by Rousseau

T1	Tumour ⩽1/3 of the circumference of the anal canal, without sphincter infiltration
T2	Tumour ⩽1/3 of the circumference of the anal canal, with sphincter infiltration
T3	
T3a	Tumour with invasion of the rectum ⩽4 cm
T3b	Tumour with invasion of the rectum >4 cm
T4	Extension to others tissues
T4a	Vagina or vulva
T4b	Other tissues
N0	No lymph node involved
N1	Unilateral inguinal lymph nodes involved
N2	Bilateral inguinal lymph nodes involved
N3	Fixed inguinal lymph nodes involved

) (Rousseau *et al*, 1973). The mean age was 65 years, and the sex ratio was nine women to one men.

### Treatments

Most patients underwent pelvic irradiation. A 4-field box technique was used. The top field was located at the L5–S1 interspace and the bottom field, 2 cm below the lowest margin of the tumour. The inguinal nodes were only covered by the anterior field. A complementary electron boost was delivered to the inguinal nodes.

The prescribed dose at the ICRU point was 50 Gy for the pelvis and 45 Gy for N0 nodes. Doses were delivered in five fractions per week, and fraction doses ranged from 1.8 to 2 Gy.

Evaluation was repeated 1 to 2 months following treatment. A boost of 15–20 Gy was delivered to responding patients using either direct perineal field, reduced 4-field, or brachytherapy. Based on clinical evaluation results, an additional 20 Gy was also delivered to all nodes involved.

The low-dose rate procedure (with 192-iridium) was used for patients who underwent brachytherapy. The prescribed dose was applied to the 85% isodose. When combined with external beam radiotherapy, the sum of the 85% reference isodose and external dose was used. Median doses of 48 and 63 Gy were delivered to the pelvis and to the anal canal, respectively (36–75).

Abdominoperineal (AP) resection was performed in patients who did not respond to a 50 Gy treatment, when residual tumour was detected after completion of the treatment, or in case of local recurrence. Colostomy alone was performed in patients with rectovaginal fistulae or severe radiation-induced complications.

Chemotherapy, consisting of a continuous infusion of 5-FU (600 mg m^−2^) and cisplatin (20 mg m^−2^) for 5 days of every 21-day cycle (J1=J21), was concomitant with radiotherapy. A total of two to three courses were given. The treatment is decided at our weekly meeting with participation of surgeons, radiation, and medical oncologists as a function of our protocols and patient's performance status, tumour and lymph node stage. Usually, we use concurrent chemotherapy and radiotherapy for T3/T4 or/and N+ patients.

### Early complications

Early complications were graded according to the NCI common toxicity criteria, version 2.

### Late complications

Late complications were graded according to the Rousseau classification system (Rousseau *et al*, 1979) (Table 3

**Table 3:** Complication grading system used

Grade I	Minor complications without treatment
Grade II	Serious complications requiring medical treatment
Grade III	Serious complications requiring surgery

). Each patient's case was analysed and discussed among two of the authors (LC and XB).

### Statistical analysis

Patients were compared in the two treatment groups with the *ϰ*^2^ test, with Fisher's exact test when necessary. For continuous variables, comparison was assessed by the Mann–Whitney test.

Survival, relapse-free interval and late complication rates were estimated by the Kaplan–Meier method, and compared by the log-rank test. Curves were calculated from the date of the histological diagnosis. The latter comparisons were preformed only in the T3/T4 patients in order to restrict patients' heterogeneity in the two treatment groups.

## RESULTS

### Factors considered in the choice of treatment

Results are summarized in Table 1. A total of 29 patients (43.3%) were treated with radiotherapy and concomitant chemotherapy. Tumour size, patient's age, and inguinal nodal status were considered in selecting treatment.

Concomitant chemotherapy was mostly (*P*<0.006) delivered to patients with higher-grade tumours, that is, 55.8% of patients with a T3/T4 tumour and 67% of patients with a tumour >4 cm in size.

**Figure 1:**
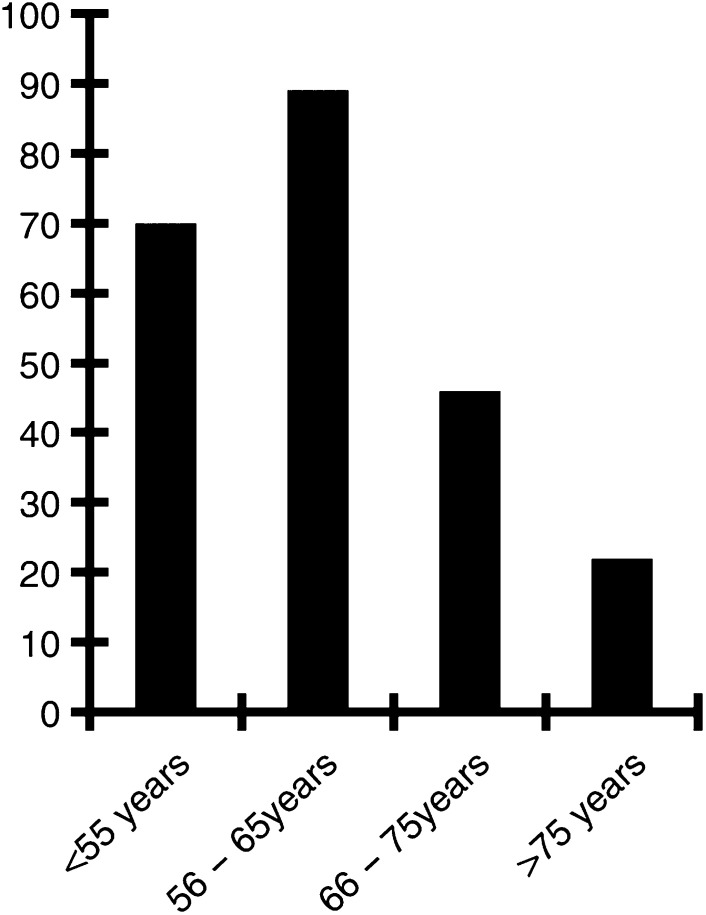
Delivery of concurrent chemotherapy and radiotherapy according to age group, in patients with T3/T4 tumours. *x*: age groups. *y*: proportion of patients who underwent concomitant chemotherapy and radiation therapy (%).

A majority of younger patients were treated with concomitant chemotherapy (*P*<0.03): 47% of patients under 56 years, 61% of patients between 56 and 65 years, 34% of patients between 66 and 75 years, and 16% patients over 75 years. For the T3/T4 patients alone (Figure 1

), these rates were 69, 90, 45, and 20%, respectively.

As regards the N status, 59% of N+ patients underwent concomitant chemotherapy and 77% for the inguinal N+.

### Survival

**Figure 2:**
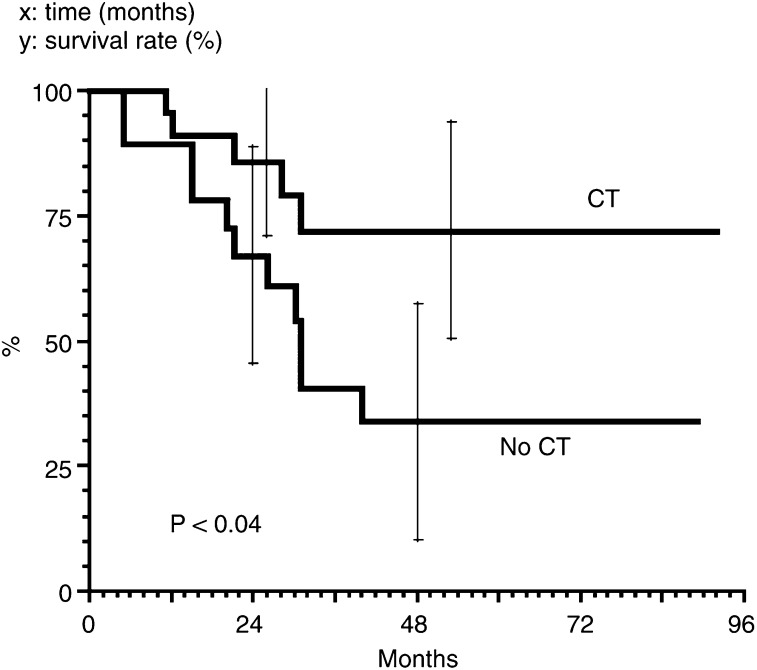
Overall survival for the T3/T4 patients. *x*: time (months). *y*: survival rate (%).

The overall 4-year survival rate was 66%. The 4-year survival rate of T3/T4 patients (Figure 2

) was 72 and 34%, with or without chemotherapy, respectively (*P*<0.04). There was, however, no significant difference in cause-specific survival at 2 years (86 *vs* 81%) and at 4 years (72 *vs* 47%).

### Relapse-free survival

**Figure 3:**
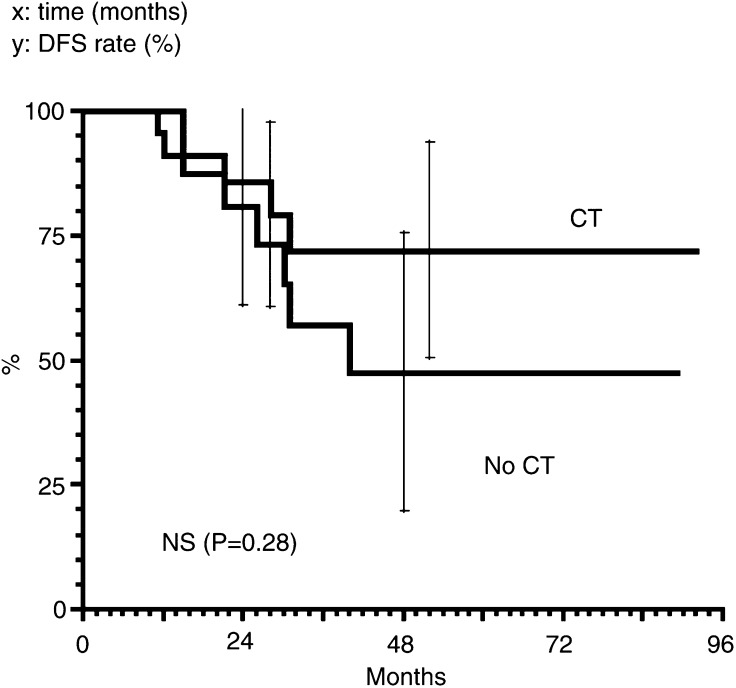
Relapse-free survival for T3/T4 patients. *x*: time (months). *y*: DFS rate (%).

The overall 4-year relapse-free survival (RFS) was 70%. The 4-year RFS rates of T3/T4 patients, with or without concomitant chemotherapy, were 78 and 60%, respectively (NS) (Figure 3

).

### Early toxicity

Increased incidence of haematological complications was observed with concurrent chemotherapy (*P*<0.001 with a *χ*^2^ test for the three ligneous). No difference in the rates of cutaneous, digestive, and renal complications was noted between both the treatments.

### Late complications

**Figure 4:**
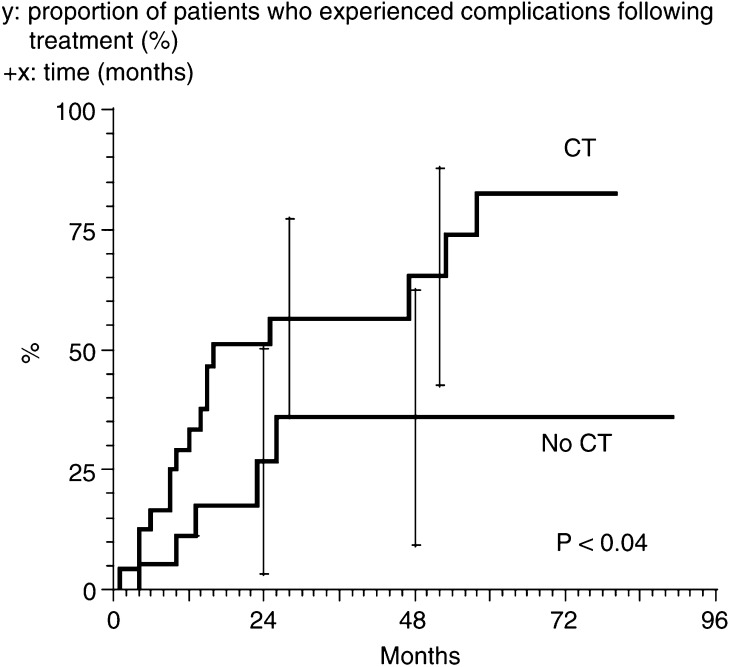
Complications observed after anal canal cancer treatment by radiation therapy alone or concurrent chemotherapy and radiation therapy. *y*: proportion of patients who experienced complications following treatment (%). +*x*: time (months).

Late complications were staged according to our own classification system (Table 3). In all, 33 patients had late complications for 39 complications – 32 patients had grade 1 complications and seven patients had grade 2/3 complications. The 2-year complication-free interval rates without or with chemotherapy were 71.5 and 54% for all the population, and 73 and 49% for the T3/T4 patients, respectively. At 4 years, the complication-free interval rates were 64 and 35%, 54 and 32.5%, respectively. The difference was significant for the T3/T4 (Figure 4

).

## DISCUSSION

As of late, concurrent chemotherapy and radiotherapy has been considered the ‘gold-standard’ of treatment for anal canal cancer. After a lot of series with different chemotherapies, 5FU and Mitomycine C (Sischy, 1985; Cummings *et al*, 1991), 5FU and Cisplatine (Rich *et al*, 1993; Doci *et al*, 1996), Bleomycine (Friberg *et al*, 1998), two articles reporting on randomised trials showed the advantages of the 5FU/Mitomycine C combination treatment in terms of local control and colostomy-free interval (UKCCCR, 1996; Bartelink *et al*, 1997). In the first series, however, this treatment was only delivered to T3/T4 and N+ patients. In the second study (UKCCCR, 1996), although all patients received radiotherapy and concomitant chemotherapy, the patient distribution showed that more than 53% of patients had a T3/T4 tumour, and close to 20% of patients had an N+ tumour. Moreover, for both series the combined treatment was restricted to younger patients.

In a previous study (Chauveinc *et al*, 2003), the majority of our patients had a UICC T1/T2 (International-Union-Against-Cancer, 1987) tumour (65%) and were over 65 years old (55%). If chemotherapy is not indicated for older patients and patients with small tumours, what would be our routine prescription for these patients?

In order to answer this question, we analysed results obtained with 67 anal canal cancer patients treated with either radiotherapy alone, or 5FU/cisplatin concurrent chemotherapy and radiation, at our institution between 1990 and 1996.

In this series, only 29 patients (43.3%) were given the combined treatment. Factors considered to determine the indication for this treatment included tumour size and T stage. The combined treatment was mostly given to patients with large-sized tumours, that is, 55.8% of T3/T4 patients and 67% of patients with a tumour ⩾4 cm in size. All of these patients should have normally had chemotherapy alone. Why were not the remainder given chemotherapy?

The main reason was their age. A larger proportion of younger patients had concurrent chemotherapy and radiation (*P*<0.03): nearly 50% of patients ⩽65 years *vs* nearly 20% of patients were over 75 years. In T3/T4 patient population, the rates were 69% of patients under 55 years, 90% of patients between 56 and 65 years, 45% of patients between 66 and 75 years, and 20% of patients over 75 years.

We naturally did not prescribe chemotherapy for the older patients, even when tumour size would have warranted chemotherapy. Early local complication rate did not seem to increase with concomitant chemotherapy; only the haematological toxicity increased. Results obtained regarding late toxicity greatly influenced our choice of treatment. We analysed late complications with our own very simple and reproducible classification system. The number of complications significantly increased after concomitant chemotherapy. Patients experienced mostly minor, but nonetheless disturbing, complications such as diarrhoea (principally), rectum haemorrhage, and anal fibrosis. Complications required medical treatment in eight patients; however, none of the patients had to undergo surgery.

The authors of the two aforementioned studies apparently obtained different results (UKCCCR, 1996; Bartelink *et al*, 1997); they failed to mention differences in complication rates. Some differences, however, could be noted. Indeed, in the first series (Bartelink *et al*, 1997) early complication rates, especially those pertaining to haematological complications, were higher in patients who had undergone concomitant chemotherapy. We do not know which classification system was used to grade complications, but it was obviously different from ours. In the second series (UKCCCR, 1996), only serious complications were analysed; in our series, we also considered grade 1 toxicity.

And the most important point is the difference of the chemotherapy used. In our series, the treatment was a 5FU/cisplatin combination, more frequently using in France for anal cancer (Gerard *et al*, 1998) and cervix cancer (Nguyen *et al*, 2002). If the early toxicity with this treatment was low, with no related death, the cisplatin could modify the late toxicity.

In another retrospective study, authors showed an increased complication rate after concurrent chemotherapy and radiotherapy (Flam *et al*, 1987). All of the severe complications were experienced by elderly patients who had undergone the combined treatment (Allal *et al*, 1997,). These results reflect our findings.

In conclusion, our decision not to treat older patients with comorbidity with concurrent chemotherapy and radiotherapy is supported by the fact that an increased late complications rate is associated to this combined treatment. Perhaps reduction of doses of chemotherapy must be discussed for this population of patients.
